# A novel biocompatible zwitterionic polyurethane with AIE effect for cell imaging in living cells†

**DOI:** 10.1039/c7ra13238g

**Published:** 2018-02-12

**Authors:** Junhuai Xu, Rui Yan, Haibo Wang, Zongliang Du, Jun Gu, Xu Cheng, Junjie Xiong

**Affiliations:** Textile Institute, College of Light Industry, Textile and Food Engineering, Sichuan University Chengdu 610065 China scuchx@163.com; Department of Pancreatic Surgery, West China Hospital, Sichuan University Chengdu 610041 China junjiex2011@126.com; Department of Cardiovascular Surgery, West China Hospital, Sichuan University Chengdu Sichuan 610041 China

## Abstract

In this work, a novel and stable zwitterionic polymer (TPE-CB PUs) was prepared to realize a cellular imaging system. TPE was conjugated into the backbone of zwitterionic polyurethane, which could be well dispersed in aqueous solution and emitted strong blue fluorescence because the TPE segment was aggregated in the core of TPE-CB PUs micelles. More importantly, the TPE-CB PUs micelles showed significant stability in a large pH window and with different storage times. In addition, the micelles exhibited low cytotoxicity in HeLa cells and mainly distributed in the cytoplasm after being incubated with cells. The outstanding properties of TPE-CB PUs combining the merits of AIE and a zwitterionic segment highlight its potential for use as a cell imaging material with remarkable capability.

## Abstract 2

In this work, a novel and stable zwitterionic polymer (TPE-CB PUs) was prepared to realize a cellular imaging system.

## Graphical abstract

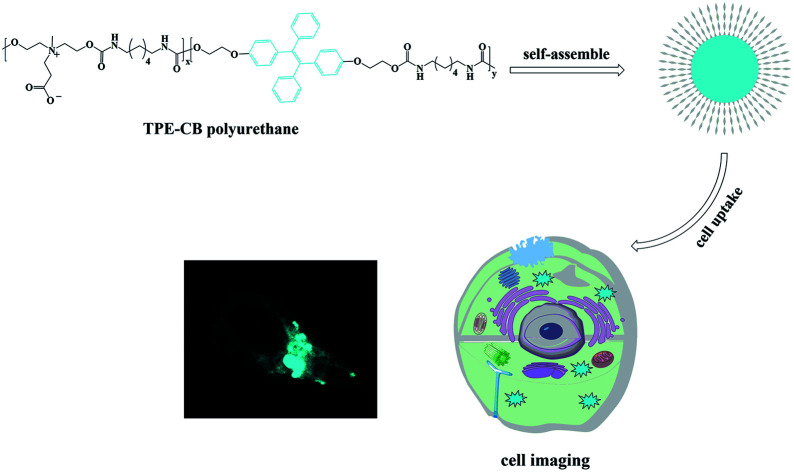

## Introduction

With the development of fluorescence microscopy techniques, cell imaging has been widely used to study cell biology and biological systems.^1–3^ Until now, many kinds of fluorescent probes including organic, inorganic or organic–inorganic hybrid materials have been applied in bio-sensing and cell imaging.^4–7^ Most of the conventional organic dyes are aromatic compounds and aromatic group stacking may lead to aggregation-caused quenching (ACQ) effects,^8^ which could impair the brightness and sensitivity in biological applications. Moreover, inorganic materials such as quantum dots usually have high cell toxicity, which greatly limits their biological applications. Therefore, development of fluorescent materials with good biocompatibility and a high signal-to-background ratio is urgently needed. Recently, a new class of fluorescent dyes with aggregation-induced emission (AIE) characteristics has been extensively used for the design of cellular imaging fluorescent probes.^9–14^ AIE behavior has attracted great attention because it can effectively avoid the ACQ encountered in conventional dyes.^15–20^ Furthermore, AIE fluorogen only light up under aggregate conditions, which give rise to exceptionally high signal-to-background ratio. Among them, tetraphenylethylene (TPE) is a typical AIE molecule.^21–28^ Once aggregated, the restriction of intermolecular rotation diminishes the non-radiative decay of the excited state energy, leading to aggregates with high quantum yields. More importantly, the TPE is very easily to modify, which make it possible to design probes based TPE for imaging of cells.

Until now, many kinds of TPE derivatives were utilized for cell imaging.^15–18,21,29,30^ However, AIE probes with better biocompatibility, biodegradability and stability is necessary for biomedical applications. Amphipathic polymer, which could self-assembly into core–shell micelles, is a promising strategy for delivering AIE molecule. In our previous study, we have synthesized polyethylene glycol (PEG) conjugated TPE and can form nanoparticles in aqueous solution, which showed low the critical micelle concentration (CMC), low cytotoxicity and photostability.^17^ PEG is the most popular antifouling materials used for biomedical application. However, it has been reported that the PEG can be oxidized by alcohol dehydrogenase, leading to lose its non-fouling capability in biological environment. Recently, zwitterionic polymers have attracted considerable attention as the alternative of PEG due to its better antifouling ability to prevent protein adsorption.^23,31–44^ Jiang's group^39,45–47^ has prepared many kinds of surfaces coated with PCBMA, PSBMA or PMPC, all the results exhibited that these surfaces have excellent antifouling properties and stability *in vivo*.

However, most of the zwitterionic materials prepared using zwitterionic methacrylate through free radical polymerization or living polymerization. Unfortunately, the backbone of these polymers was non-biodegradable and the materials could not be eliminated through metabolism. It compels us to pursue for new method to synthesize zwitterionic polymers. Polyurethane (PU) is known to be one of the most versatile polymers. The structure and properties of PU can be tailored by varying the constituents of these hard and soft domains.^48,49^ Moreover, PU is a well-established biodegradable polymer and its biocompatibility makes it suitable for application *in vivo*.

**Scheme 1:**
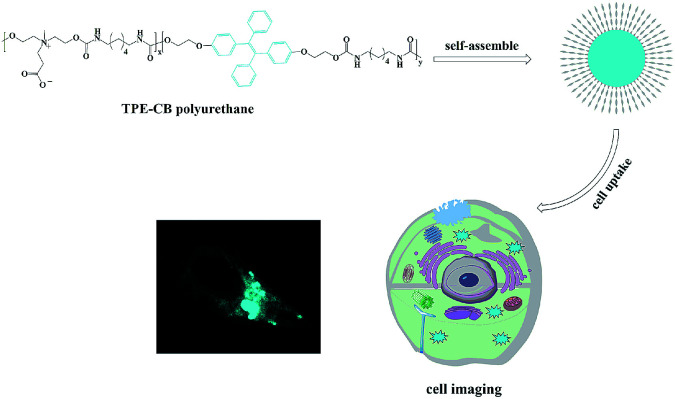
Illustration of TPE-CB PUs micelles with AIE properties for intracellular imaging.

In this contribution, zwitterionic monomer (dihydroxy carboxybetaine, DHCB), hexamethylene diisocyanate (HDI) and TPE-based monomer (dihydroxy TPE-based, HO-TPE-OH) were condensed *via* polyaddition reaction to construct an amphiphilic copolymer with AIE fluorescence. As shown in Scheme 1, HO-TPE-OH, HDI and DHCB were polymerized to form the amphiphilic polyurethane. The obtained amphiphilic polyurethane is inclined to self-assembly into ultra-stable TPE-CB PUs micelles and could well disperse in water and physiological solution. And then, a series of characterization methods, including gel permeation chromatography (GPC), UV-visible absorption spectroscopy, Fourier transform infrared spectroscopy (FT-IR), fluorescence spectroscopy, transmission electron microscopy (TEM), and dynamic light scattering (DLS) were used to characterize the properties of TPE-CB PUs and micelles. Furthermore, the biocompatibility and cell uptake behaviors of TPE-CB PUs micelles were investigated to evaluate the biology performance of this material.

## Experiments

### Materials

Hexamethylene diisocyanate (HDI), diethanolamine, iodomethane, 2-bromoethanol, trifluoroacetic acid (TFA) were purchased from Adamas. Zinc powder, 4-hydroxybenzophenone, TiCl_4_, *tert*-butyl acrylate and KI, K_2_CO_3_ were acquired from Aladdin. Other solvents and reagents all came from commercial sources and were used as received without further purification. Deionized water was used in all process of the experiments.

### Instrumentations

^1^H nuclear magnetic resonance (NMR) spectra were recorded on Bruker AV 400 spectrometers (400 MHz, Germany) with deuterated solvents (chloroform-D, dimethyl sulfoxide-D6 and methanol-D4). Mass spectrometry was used to confirm the molecular weight. UV-visible absorption spectra were measured by Analytic-jena Specord S600 (Germany) spectrophotometer in absorbance mode using quartz cuvettes of 1 cm path length. Fourier transform infrared spectroscopy was detected by a Shimadzu Spectrum 8400 spectrometer (Japan) in diffuse reflectance mode. GPC analyses of polymers were performed by HLC-8320GPC and using DMF as the eluent. Perkin Elmer LS-55 spectrofluorometer was used to measure the fluorescence spectra. The excitation wavelength was set at 350 nm, with a slit width of 10 nm. The size distribution of the micelles in water was determined by Zetasizer Nano S90 from Malvern Instruments equipped with a He–Ne laser at a wavelength of 633 nm at 25 °C. The samples were filtered by a 0.45 μm millipore before measurement. To amplify the micelles, TEM images were conducted on a JEM-1200EX microscope operated at 100 kV, the TEM specimens were made by placing a drop of the micelles solution on a carbon-coated copper grid.

### Synthesis of dihydroxy carboxybetaine (DHCB)

Diethanolamine (10.5 g, 0.1 mol) and *tert*-butyl acrylate (12.8 g, 0.1 mol) were dissolved in a flask with methanol (35 mL). After 48 hours' reaction at room temperature, the methanol was removed under reduced pressure to obtain the crude product, and then the crude product was purified by silica gel chromatography with dichloromethane (DCM) and methanol (MeOH) as eluent (DCM : MeOH = 95 : 5, v/v) and a colorless oil (A) was obtained.

*N*-Propionate-diethanolamine (11.65 g, 0.5 mol) and iodomethane (14.2 g, 0.5 mol) were dissolved in a 100 mL flask with acetonitrile (35 mL). The reaction was held at room temperature for 48 hours, the acetonitrile was then removed under reduced pressure to obtain the product B (yellow oil). Its chemical structure was confirmed by ^1^H NMR in methanol-D4 on a 400 MHz spectrometer.

This produce B was added into a solution (DCM : TFA = 1 : 1, v/v) and stirred vigorously for 3 hours, the mixture was added into anhydrous ether drop by drop for two times and a yellow oil product C was obtained from vacuum drying (the detailed synthetic route see Scheme S1†).

### Synthesize of dihydroxy TPE-based (HO-TPE-OH)

To synthesize HO-TPE-OH (Scheme S2†), first is to synthesize 2-(4-benzoylphenoxy)ethan-1-ol. 4-Hydroxybenzophenone (19 g, 0.1 mol), 2-bromoethanol (12.5 g, 0.1 mol) and K_2_CO_3_ (27.6 g, 0.2 mol) were dissolved in a 250 mL flask with a reflux condenser at 110 °C for 24 hours. After cooling the mixture to room temperature, 150 mL deionized water was added and extracted with DCM for five times. The crude product was purified by a silica gel column using a mixture of petroleum ether : ethyl acetate (10 : 1, v/v) and a white crystalline solid (D) was obtained. In the second step, D (48.4 g, 0.1 mol), zinc powder (16 g, 0.24 mmol) and 300 mL THF were added into a three-necked flask equipped with a magnetic stirrer at 0 °C with an N_2_ atmosphere. Then, TiCl_4_ (13 mL, 0.12 mmol) was slowly added with the temperature under 5 °C. The mixture stirred at the room temperature for overnight. After this, 200 mL hydrochloric acid (1 mmol L^−1^) was added and extracted with DCM for three times. The crude product was purified by a silica gel column and evaporated under vacuum to get product (E) as a fallow solid. The obtained fallow solid was HO-TPE-OH and its chemical structure was confirmed by ^1^H NMR in CDCl_3_ on a 400 MHz spectrometer and mass spectrometry.

### Synthesize of TPE-CB PUs

The TPE-based polyurethane was synthesized according to a standard procedure. Briefly, amount 0.5 g DHCB was placed in a 50 mL round-bottom flask with a stir bar at 105 °C for 2 hours to remove any trace of water in the system through reduced pressure. And then cooled to room temperature, 0.897 g HDI was added into the flask, and 5 mL DMF was added to dissolve them, then 0.0002 g catalyst dibutyltin dilaurate was injected sequentially. Temperature was then raised to 85 °C, after 4 hours of polymerization under a nitrogen atmosphere, 0.5 g HO-TPE-OH was added to the mixture as the chain extender, after that 2 mL methanol was added to terminate with the residual isocyanate group. The reaction mixture was precipitated in cold anhydrous ether. Fallow polyurethane (TPE-CB PUs) was collected through filtration followed by drying under vacuum. ^1^H NMR and infrared spectrum were used to confirm its chemical structure.

### Preparation of TPE-CB PU micelles

TPE-CB PUs micelles were prepared by a dialysis method. In brief, TPE-CB PUs (10 mg) was dissolved in a mixture of methanol (2 mL) and DMSO (2 mL). Afterwards, 10 mL deionized water was dropwise added under vigorous agitation, continued for 3 hours. The generated solution was transferred into a dialysis bag (MWCO = 3500 Da) and dialyzed against deionized water for two days to remove any residual solvent. Subsequently, the solution was filtered through 0.45 μm pore-sized microporous membrane.

Dimensions of micelles were determined by DLS. A Malvern Z90 Zetasizer equipped with a 633 nm He–Ne laser and an avalanche photodiode detector was provided to characterize the hydrodynamic size of the self-assemblies. With the room temperature, the scattering light at an angle of 90° was detected and used to analyse the size and distribution. Additionally, the morphology of TPE-CB PUs micelles was observed by TEM.

### *In vitro* cell cytotoxicity assay

The cell viability of TPE-CB PUs micelles and HO-TPE-OH solution on HeLa cells was evaluated by cell counting kit-8 (CCK-8) assay, respectively. Briefly, cells were seeded in 96-well microplates at a density of 1.5 × 10^5^ cells per mL in 100 μL media containing 10% FBS, respectively. After cells attachment for 24 hours, the cells were cultured with 10, 50, 100, 200, 500 μg mL^−1^ TPE-CB PUs micelles and HO-TPE-OH solution for 48 hours. Then PBS was used to wash all samples for a few times to remove the free materials. 10 μL of CCK-8 dye and 100 μL of DMEM cell culture medium were added to each well and incubated for 4 hours at 37 °C. Plates were then analysed with a microplate reader (Thermo Multiskan GO). The formazan dye absorbance was measured at 450 nm, with the reference wavelength at 600 nm. The number of live cells was proportional to the values. The percent reduction of CCK-8 dye was compared to controls (cells not exposure to TPE-CB PUs and HO-TPE-OH), which represented 100% CCK-8 reduction. Three replicate wells were used per microplate, and the experiment was repeated three times. Cell survival was expressed as absorbance relative to that of untreated controls. Results are presented as mean ± standard deviation (SD).

### Confocal microscopic imaging

HeLa cells were cultured in Dulbecco's modified Eagle's medium (DMEM) supplemented with 10% heat-inactivated FBS, 2 mM glutamine, 1% penicillin, and 1% of streptomycin. Cell culture was maintained at 37 °C in a humidified condition of 95% air and 5% CO_2_ in culture medium. Culture medium was changed every 3 days to maintain the exponential growth of the cells. A day before treatment, cells were seeded in 6-well microplates with a density of 1 × 10^5^ cells per dish. Then, the cells were incubated with TPE-CB PUs micelles at a certain concentration of 50 μg mL^−1^ for 3 hours at 37 °C. Afterward, the cells were washed three times with PBS to remove the TPE-CB PUs micelles and then fixed with 4% paraformaldehyde for 10 min at room temperature. Cell images were taken with a confocal laser scanning microscope (CLSM) Zeiss 710 3-channel (Zeiss, Germany) with the excitation wavelength of 350 nm.

## Results and discussion

### The synthesis of TPE-CB PUs

**Scheme 2:**
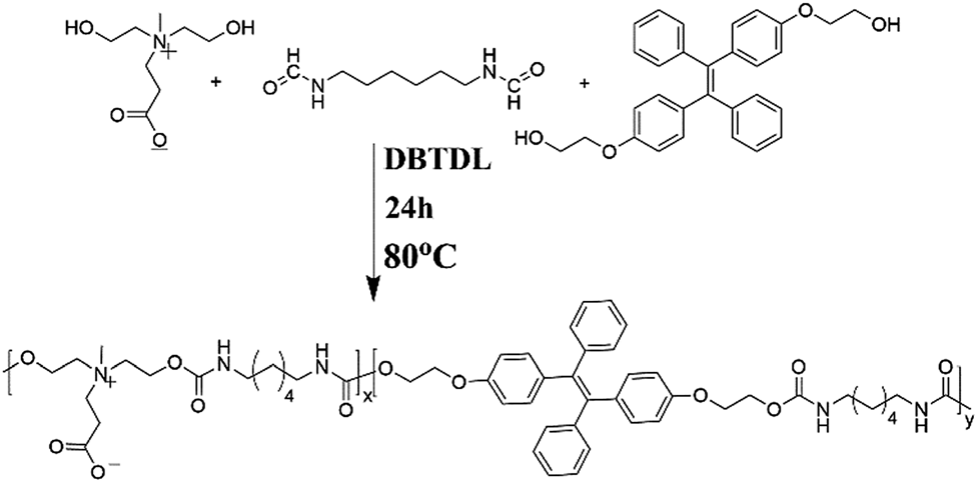
Detailed synthetic route of the TPE-CB PUs.

TPE is an iconic AIE fluorogen: it is fluorescence free in good solvents, but becomes emissive in a poor solvent. The simple structure makes it an ideal model for construction of various fluorescent materials. Therefore, TPE-based polyurethane micelles with zwitterionic shell possess great potential for various bio-applications. In this article, TPE-CB PUs was synthesized by a simple polymerization. The synthesis routes were shown in Scheme 2.

To start, the isocyanate-terminated zwitterionic pre-polymers were prepared, and then HO-TPE-OH was added as a chain extender. The zwitterionic monomer was illustrated in Fig. S1.† And the ^1^H NMR spectra of HO-TPE-OH were illustrated in Fig. S2.† The major proton peaks of zwitterionic monomer and HO-TPE-OH in the ^1^H NMR spectra were marked and conformed well to the molecular structure. The mass spectra were further confirmed the structure of HO-TPE-OH (as shown in Fig. S3†), the *m*/*z* is located at 452.2. And the TPE-CB PUs was also confirmed by ^1^H NMR and FT-IR.

**Fig. 1:**
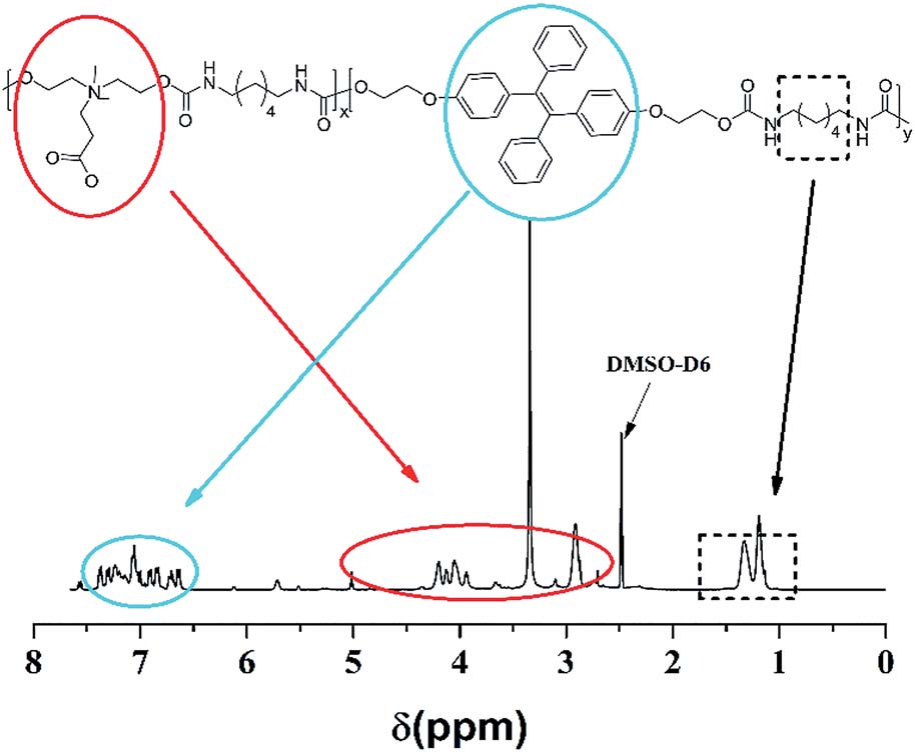
^1^H NMR spectra of the TPE-CB PUs in DMSO-D6.

As shown in Fig. 1, the signals located in the circle of blue were attributed to the protons of TPE segments; in the red circle were assigned to the protons of DHCB segments; in the black circle belonged to the alkane chains. Compared the HO-TPE-OH and DHCB with TPE-CB PUs, we could observe these chemical shifts belonged to them are shown in the spectra of TEP-CB PUs, demonstrating the successful synthesis of TPE-CB PUs. Besides, the FT-IR spectroscopy was used to investigate the chemical composition and structure of TPE-CB PUs. As shown in Fig. S4,† the characteristic peaks at 1701 cm^−1^ and 1535 cm^−1^ were attributed to –NH–COO– of TPE-CB PUs. The peaks at 3331 cm^−1^, 2920 cm^−1^ and 2864 cm^−1^ belonged to the stretching vibrations of –NH_2_ and –CH_2_. As a consequence, based on the ^1^H NMR and FT-IR spectra, we could perorate that the TPE-CB PUs were synthesized triumphantly. Additionally, the deformation vibration of Ar-H located at 758 cm^−1^ and the stretching vibration of benzene skeleton vibration located at 1590 cm^−1^ were observed in the sample of TPE-CB PUs, suggesting successfully incorporation of TPE to afford TPE-CB PUs. Moreover, the molecular weight and polydispersity distribution of TPE-CB PUs was measured by GPC. The number molecular weight was 8.9 kDa with the polydispersity index (*M*_w_/*M*_n_) of 1.86.

### Self-assembly of TPE-CB PUs micelles

**Fig. 2:**
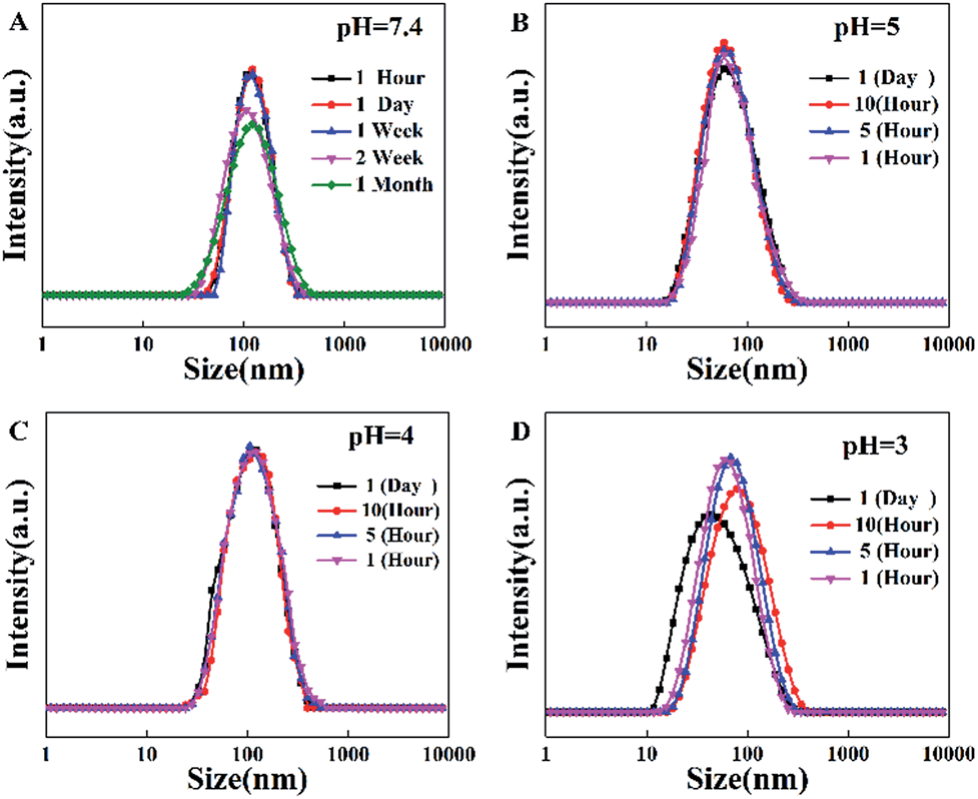
(A) The diameter of TPE-CB PUs micelles in pH = 7.4, (B) diameter in pH = 5, (C) diameter in pH = 4, (D) diameter in pH = 3.

TPE-CB PUs contains hydrophobic segments (HO-TPE-OH) and hydrophilic segments (DHCB). Therefore, the micelles could be easily formed in the aqueous solution with hydrophilic DHCB as the zwitterionic shell. While, the hydrophobic TPE was encapsulated in the core, leading to the enhanced quantum yield of the micelles. To confirm the TPE-CB PUs could self-assemble into micelles in aqueous solution, the size of the TPE-CB PUs was measured by DLS. As shown in Fig. 2A, the diameter of the TPE-CB PUs micelles was 112 nm with a narrow size distribution in PBS buffer (pH 7.4). Even the micelles solution was stored for 1 month, the size was changed slightly. Furthermore, the stability of the micelles under other pH was also studied. The results indicated that the polymer micelles were very stable under pH 4 or 5 (Fig. 2B and C). When the micelles solution became more acidic (Fig. 2D), the size was not very stable, and the size was changed obviously with the prolonged storage time. The polymer micelles were stable in a large pH window. The main reason may be attributed to the protein-resistant zwitterionic shell. It could be considered to suit for the goal of long-term cellular tracing and even the further physiological applications.

**Fig. 3:**
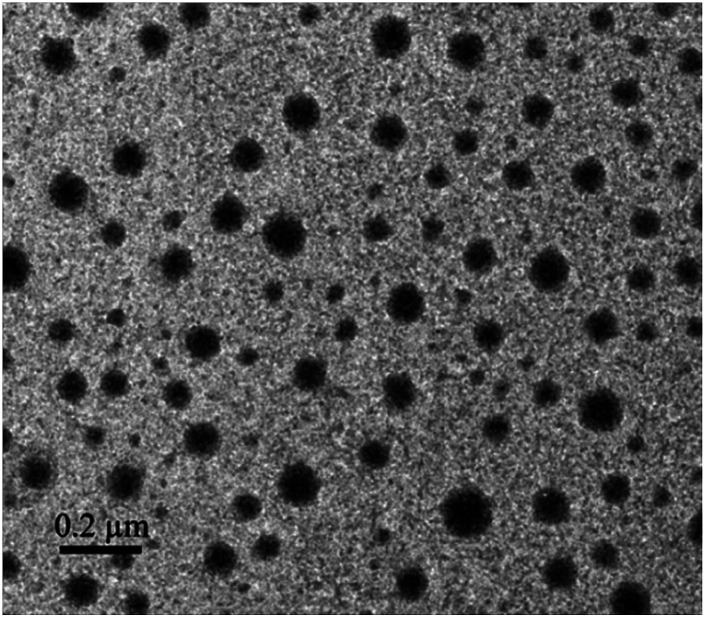
TEM image of TPE-CB PUs micelles.

The morphology of the micelles was further investigated by TEM (Fig. 3), which showed that the micelles were spheroidal and equally distributed, and the diameter is about 50–70 nm. The above results that smaller than what from the DLS were ascribed to the evaporation of water.

### Fluorescent behaviour of TPE-CB PUs and micelles

**Fig. 4:**
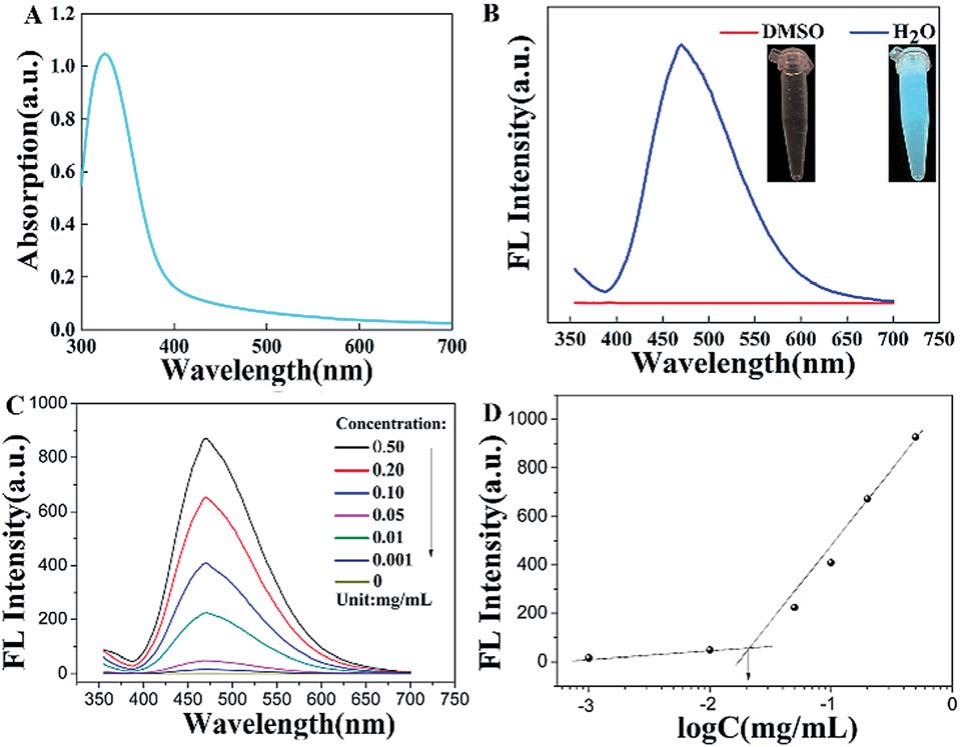
(A) The UV-vis absorption of TPE-CB PUs micelles, (B) the fluorescence intensity of TPE-CB PUs micelles in DMSO and H_2_O, (C) the fluorescence intensity of TPE-CB PUs micelles in different concentration, *λ*_ex_ = 350 nm (D) the CMC value of TPE-CB PUs micelles.

The fluorescence property is one of the important parameters to confirm whether the TPE-CB PUs micelles could be used in cell imaging. As displayed in the Fig. 4A, the maximum absorption wavelength was located at 338 nm, the UV-vis absorption spectra supported the aggregation state of the TPE-CB PUs micelles.^17,22^ In addition, as observed in the inset of Fig. 4B, the TPE-CB PUs solution have no fluorescence in DMSO under UV light of 365 nm. However, when the TPE-CB PUs was dispersed in water, the solution exhibited intense fluorescence. The further AIE effect of the TPE-CB PUs was also studied by PL spectra. The AIE characteristics of HO-TPE-OH and TPE-CB PUs are shown in Fig. S5.† The PL spectra of the polymer with different concentrations were also measured (Fig. 4C). The result exhibited that the fluorescence intensity increased with increasing concentration of TPE-CB PUs. Through the fluorescence intensity of the TPE-CB PUs under different concentration, the CMC value was determined.

The CMC value (12.6 μg mL^−1^, Fig. 4D) is much lower than some other copolymers, suggesting the TPE-CB PUs system is thermodynamically stable. All the results demonstrated that this material has excellent fluorescence properties and stability in aqueous solution. Thus, it was believed that TPE-CB PUs is highly attractive for cell imaging applications.

### *In vitro* cell cytotoxicity assay and cell imaging

**Fig. 5:**
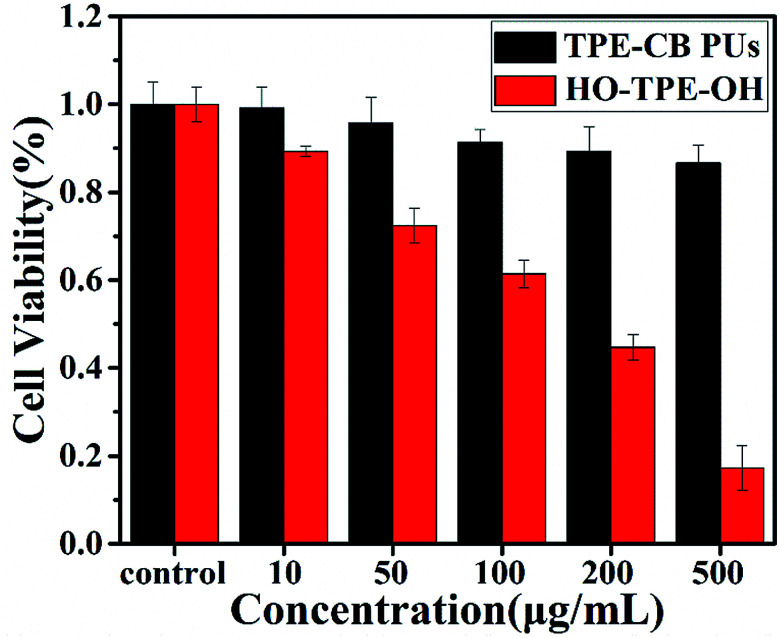
Cell viability of HeLa cells incubated with various concentrations of TPE-CB PUs micelles and HO-TPE-OH for 48 h.

Biocompatibility evaluation is an indispensable step for biomedical applications of materials. The cytotoxicity of material was analysed by the CCK-8 assay. In this work, HeLa cells were chosen as the models for cell tracking. As shown in Fig. 5, the different concentrations of the TPE-CB PUs micelles and HO-TPE-OH solution cytotoxicity had relatively large differences. From the figure, the TPE-CB PUs micelles showed quite low cytotoxicity, even at fairly high concentrations up to 500 μg mL^−1^, indicating the well cell compatibility of the prepared materials. On the contrary, an obviously result shown in the figure, HO-TPE-OH solution was much toxicity than that modified by PUs. The excellent cytocompatibility was mainly due to the fact that the zwitterionic segments (DHCB), which have great potential in the biomaterials. Based on the cells viability results, TPE-CB PUs are considered to be promising candidates for cell imaging applications due to their biocompatibility and fluorescence properties.

**Fig. 6:**
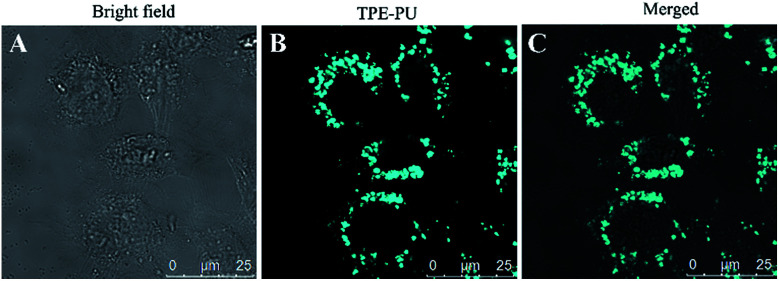
CLSM images of HeLa cells incubated with 50 μg mL^−1^ of TPE-CB PUs micelles for 4 hours. (A) Bright field image, (B) excited with 350 nm laser, (C) merged image of (A) and (B).

To explore the TPE-CB PUs micelles for bio-imaging application, HeLa cells was incubated with the micelles for 4 hours and observed by confocal laser scanning microscopy (CLSM). As shown in Fig. 6, it could observe clear and strong blue fluorescence of TPE which indicated that the micelles internalized into the cells by the endocytosis process. And then, compared with the cells in bright field and stained with the micelles, the retain of TPE-CB PUs micelles were mainly located in the cytoplasm region, indicating that TPE-CB PUs could act as fluorescent probes for intracellular imaging and other bio-applications.

## Conclusion

In summary, a novel and stable zwitterionic polymer with TPE was designed to realize a cellular imaging system. TPE was conjugated into the backbone of polyurethane, which could be easily self-assemble into micelles and display an excellent fluorescence in the aqueous solution. They can be well dispersed in aqueous solution and emitted strong blue fluorescence due to the TPE segment was aggregated in the core of TPE-CB PUs micelles. More importantly, the TPE-CB PUs micelles showed significant stability in a large pH window and with different storage times. Thus, the micelles showed a great potential in bio-imaging and physiological applications. In addition, the micelles exhibited low cytotoxicity in HeLa cells and mainly distributed in the cytoplasm after incubated with cells. The outstanding properties of TPE-CB PUs combined the merits of AIE and zwitterionic segment highlight its potential to use as a remarkable capability of cell imaging materials.

## Conflicts of interest

There are no conflicts to declare.

## Supplementary Material

RA-008-C7RA13238G-s001
