# Supplementary material for: A novel biocompatible zwitterionic polyurethane with AIE effect for cell imaging in living cells
Source: RSC Adv. 2018 Feb 12;8(13):6798–804. doi: 10.1039/c7ra13238g (PMC9078316; doi:10.1039/c7ra13238g)
Supplement: RA-008-C7RA13238G-s001 [file RA-008-C7RA13238G-s001.pdf]

Electronic Supplementary Material (ESI) for RSC Advances.
This journal is © The Royal Society of Chemistry 2018

Supporting Information(SI)

A novel biocompatible zwitterionic polyurethane with AIE effect for cell imaging in living cells†

Junhuai Xu[a], Rui Yan[a], Haibo Wang[a], Zongliang Du[a], Jun Gu[c], Xu Cheng[a*], Junjie Xiong[b*]
[a] Textile Institute, College of Light Industry, Textile and Food Engineering, Sichuan University, Chengdu, 610065, China. E-mail: scuchx@163.com
[b] Department of Pancreatic Surgery, West China Hospital, Sichuan University, Chengdu 610041, China. E-mail: junjiex2011@126.com
[c] Department of Cardiovascular Surgery, West China Hospital, Sichuan University, Chengdu, Sichuan 610041, China

Synthesize of dihydroxy carboxybetaine (DHCB)

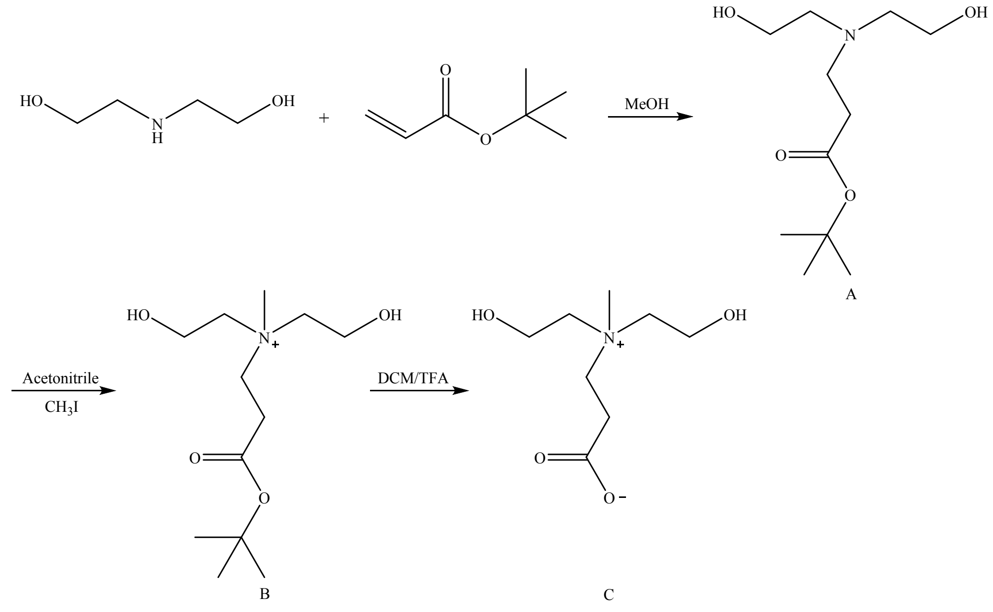

Scheme S1 the detailed synthetic route of DHCB.

Synthesize of HO-TPE-OH

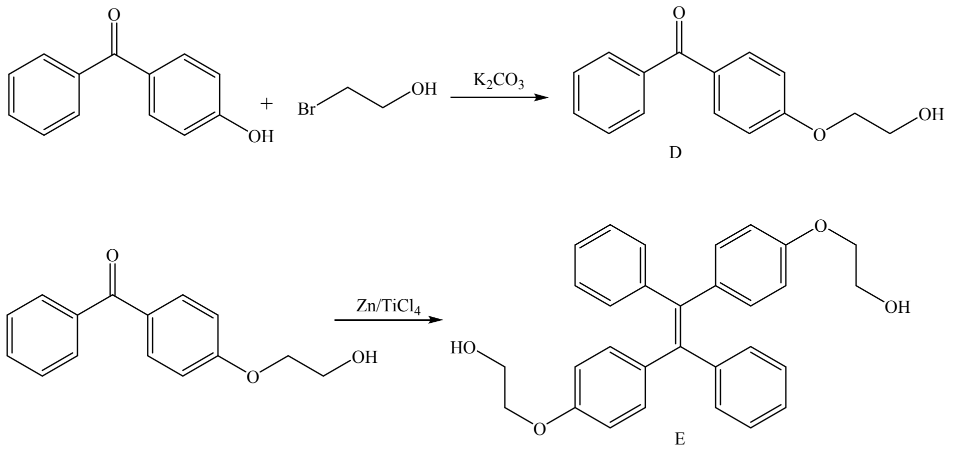

Scheme S2 the detailed synthetic route of HO-TPE-OH.

The ^{1}H NMR of tBu-DHCB

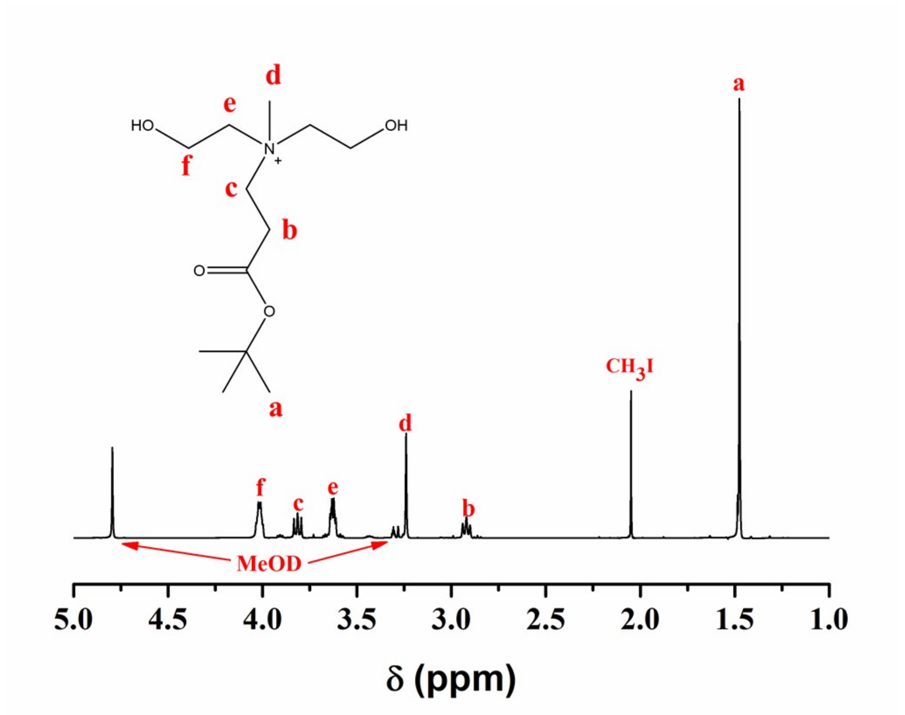

Figure S1 ^{1}H NMR of tBu-DHCB in methanol-D4

The ^{1}H NMR of HO-TPE-OH

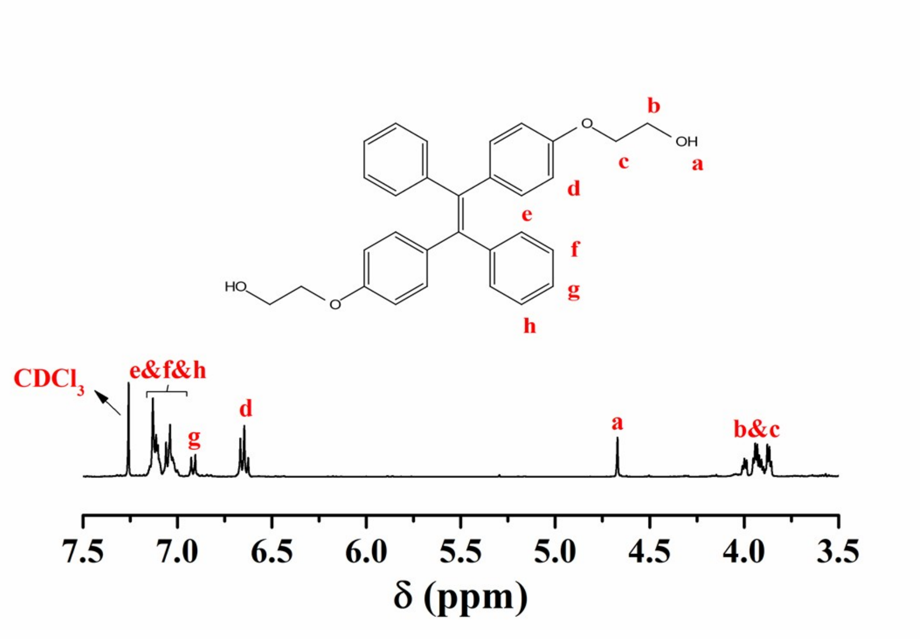

Figure S2 ^{1}H NMR of HO-TPE-OH in $CDCl_3$.

The mass spectrometry of HO-TPE-OH

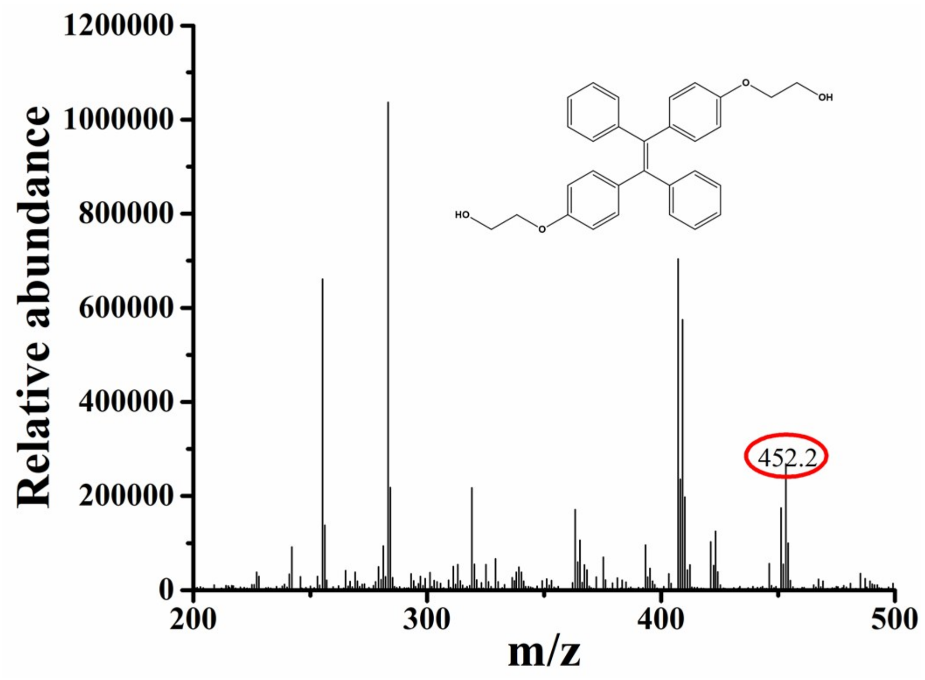

Figure S3 the mass spectrometry of HO-TPE-OH.

The FT-IR spectra of TPE, CBPUs and TPE-CB PUs

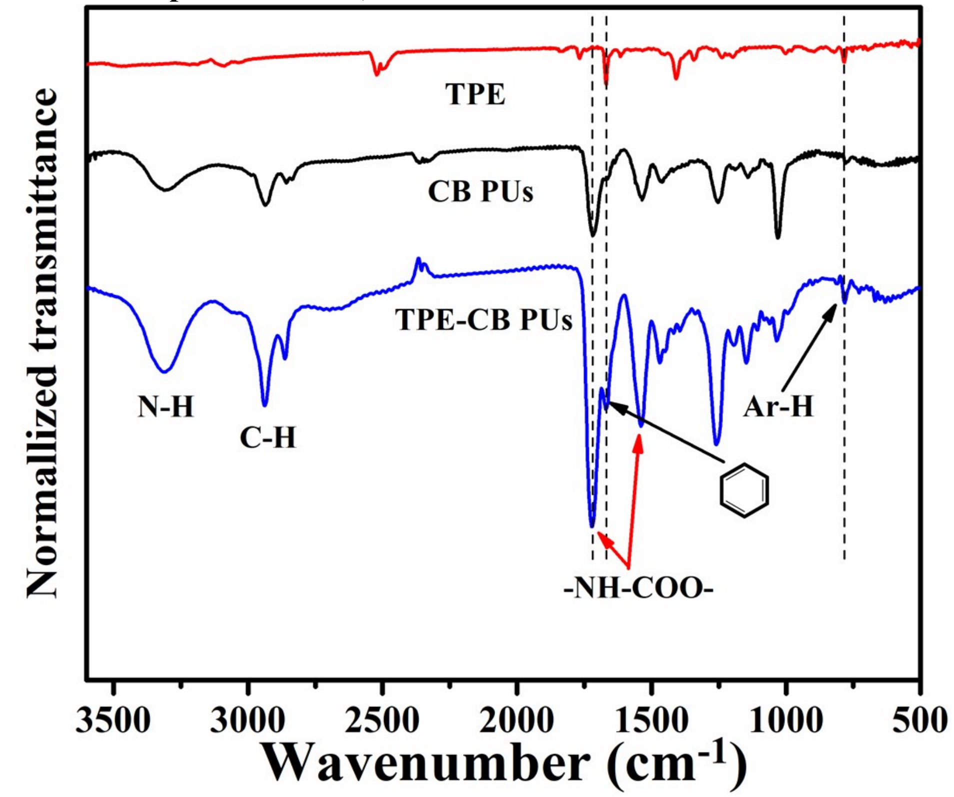

Figure S4 FT-IR spectra of TPE, CBPUs and TPE-CB PUs.

The AIE characteristic of TPE-CB PUs

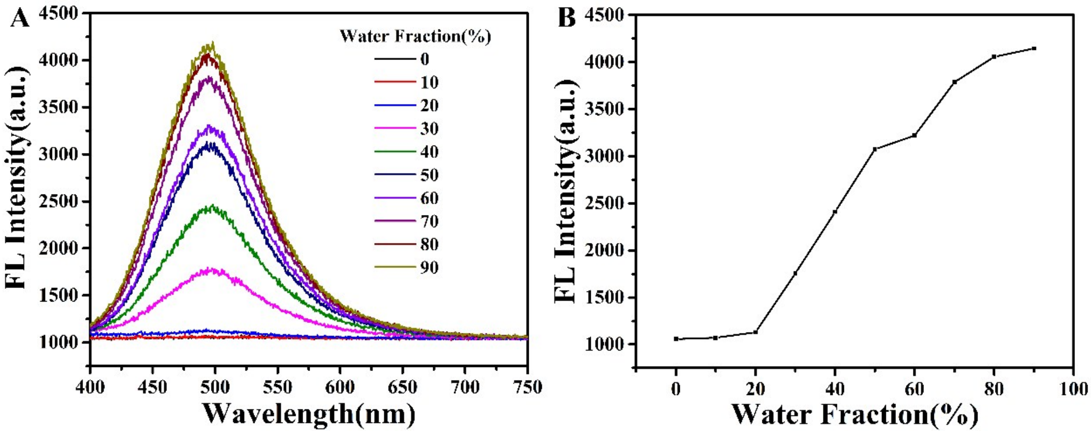

Figure S5 (A) PL spectra of TPE-CB PUs in DMSO-water mixtures with different water fraction (λ_{Ex}=350 nm); (B) the changes in PL peak of the compound in different water fraction mixtures.
